# Wave polarization and dynamic degeneracy in a chiral elastic lattice

**DOI:** 10.1098/rspa.2019.0313

**Published:** 2019-12-18

**Authors:** G. Carta, I. S. Jones, N. V. Movchan, A. B. Movchan

**Affiliations:** 1 Liverpool John Moores University, Mechanical Engineering and Materials Research Centre, Liverpool L3 3AF, UK; 2 University of Liverpool, Department of Mathematical Sciences, Liverpool L69 7ZL, UK

**Keywords:** chiral elastic lattice, lattice flux and circulation, vortex waveforms, wave polarization

## Abstract

This paper addresses fundamental questions arising in the theory of Bloch–Floquet waves in chiral elastic lattice systems. This area has received a significant attention in the context of ‘topologically protected’ waveforms. Although practical applications of chiral elastic lattices are widely appreciated, especially in problems of controlling low-frequency vibrations, wave polarization and filtering, the fundamental questions of the relationship of these lattices to classical waveforms associated with longitudinal and shear waves retain a substantial scope for further development. The notion of chirality is introduced into the systematic analysis of dispersive elastic waves in a doubly-periodic lattice. Important quantitative characteristics of the dynamic response of the lattice, such as lattice flux and lattice circulation, are used in the analysis along with the novel concept of ‘vortex waveforms’ that characterize the dynamic response of the chiral system. We note that the continuum concepts of pressure and shear waves do not apply for waves in a lattice, especially in the case when the wavelength is comparable with the size of the elementary cell of the periodic structure. Special critical regimes are highlighted when vortex waveforms become dominant. Analytical findings are accompanied by illustrative numerical simulations.

## 1. Introduction

Elastic lattices are relatively simple systems that exhibit many interesting dynamic properties, such as wave dispersion, filtering and dynamic anisotropy [1–3]. Due to their discrete nature, lattice models allow the answer to fundamental questions on dynamic fracture problems, concerning in particular the analytical prediction of the speed of crack propagation and the explanation of crack tip instabilities [4–9], that cannot be addressed by using continuum models. Homogenization theories for discrete systems based on asymptotic techniques have been applied both in the static [10–13] and in the dynamic [14–17] regimes.

Polarization of elastic waves in continuous media is well studied (see, for example, [18–20]). Recently, comparative analysis of polarization of elastic waves in a continuum versus discrete medium has been performed in [21]. It is well known that in a two-dimensional homogeneous isotropic infinite continuum two types of waves can propagate at different speeds, namely shear and pressure waves. In the former (or latter) case, the displacement vector is perpendicular (or parallel) to the wavevector. A triangular lattice approximates an isotropic continuum in the long wavelength limit or, equivalently, when the modulus of the wavevector tends to zero. For large values of the modulus of the wavevector, waves generally cannot be classified as shear or pressure waves. In [21] it was shown that there are directions corresponding to mirror symmetries where the waves are longitudinally or transversely polarized. In [21] two new quantities have been introduced, denoted as ‘lattice flux’ and ‘lattice circulation’, to characterize waves in the triangular lattice for any value of the wavevector. A decomposition of the displacement field has been proposed, whereby waves are described as a combination of flux-free and circulation-free components.

In this paper, we study a triangular lattice connected to a system of gyroscopic spinners. In this case, the trajectories of the lattice particles are not straight lines as in a classical triangular lattice, but ellipses. In some limit cases, discussed in depth in this work, the ellipses become circles. This special type of wave will be referred to as a ‘vortex waveform’.

Throughout the present paper, we will refer to the triangular lattice connected to gyroscopic spinners as a ‘chiral lattice’. According to the original definition by Lord Kelvin [22], an object is chiral if it cannot be superimposed onto its mirror image. The gyro-elastic lattice considered here is an ‘active chiral’ medium, in which chirality is brought by the action of the gyroscopic spinners on the lattice particles. This type of chirality is different from the ‘geometrical chirality’ discussed in [23–26] or from the interfacial wave guiding [27]. Chirality discussed here can be used in unidirectional wave steering, as in [28,29], to create topological insulators.

The first model of an active chiral lattice was introduced in [30], where both a monatomic and a biatomic triangular lattice attached to a uniform system of gyroscopic spinners were studied. Furthermore, the homogenized equations of the discrete system were used to model a gyroscopic continuum, that was used to design a cloaking device. The monatomic gyro-elastic lattice proposed in [30] was investigated in depth in [31], with special emphasis on tunable dynamic anisotropy and forced motions. Gyroscopic spinners were also employed to create localized waveforms in [32] and in topological protection applications in [33,34]. A hexagonal array of gyroscopes suspended by springs and magnetically coupled was built in [35], where unidirectional edge waves were experimentally observed.

Systems embedding gyroscopic spinners have many applications, especially in aerospace engineering [36–40]. For this reason, the theory of gyro-elastic continua has been developed in the literature (see, for example, [41,42]). Recently, attaching gyroscopic spinners to elastic beams in order to modify the dynamic properties of the beams has been proposed in [43,44] and creating novel low-frequency resonators for seismic applications has been discussed in [45].

The present paper is organized as follows. In §2, the governing equations and the dispersion relation for a triangular lattice connected to a system of gyroscopic spinners are reviewed. In addition, the definitions of lattice flux and lattice circulation introduced in [21] are discussed. In §3, a decomposition of the displacement field in the chiral system is introduced. Moreover, lattice flux and lattice circulation are used to fully characterize waves propagating in the medium. The analysis is performed for the triangular chiral lattice studied in this paper; however, a similar formulation can be developed for any other type of gyro-elastic lattice, once the corresponding lattice flux and lattice circulation are derived. In §4, the motion of the lattice for characteristic values of the wavevector is described. In particular, we show examples of vortex waveforms. In §5, the dynamic properties of the discrete system for limit values of the parameter characterizing the spinners are investigated using asymptotic analyses. Finally, in §6, concluding remarks are provided.

## 2. Governing equations and definitions

**Figure 1.**
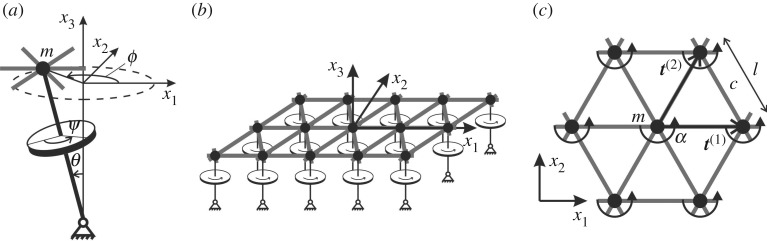
(*a*) Representation of a gyroscopic spinner, where *ψ*, *ϕ* and *θ* are the angles of spin, precession and nutation, respectively; (*b*) triangular elastic lattice connected to a system of gyroscopic spinners; (*c*) elementary cell of the lattice.

We study an infinite, periodic triangular lattice of particles with mass *m*, connected by linear springs of stiffness *c*, length *l* and negligible density. Each lattice particle is attached to a gyroscopic spinner (figure 1*a*), characterized by the spinner constant *α*, which is a function of the geometry of the spinner [30]. The lattice is shown in figure 1*b* and its elementary cell is presented in figure 1*c*. We assume that the effect of gravity is negligible and the nutation angles *θ* of the spinners are small, so that the particles move in the *x*_1_*x*_2_-plane. This is the model system introduced in [30,31].

### (a) Dispersion properties of the chiral lattice

In the time-harmonic regime, the displacement of a lattice particle ***u***(***x***, *t*) = ***U*** (***x***)e^i*ωt*^, where ***x*** = (*x*_1_, *x*_2_)^T^ is the position vector, *t* is time and *ω* is the radian frequency. The displacements of the particles of the infinite periodic lattice are assumed to satisfy the Bloch–Floquet conditions:

2.1 $$U(x+n_{1}t^{\left(1\right)}+n_{2}t^{\left(2\right)})=U(x)e^{i\;k\cdot Tn}.$$

Here, ***n*** = (*n*_1_, *n*_2_)^T^ is the multi-index, ***t***^(1)^ = (*l*, 0)^T^ and $t^{\left(2\right)}={\left(l/2,\sqrt{3}\;l/2\right)}^{T}$ are the lattice vectors (figure 1*c*) and $k={\left(k_{1},k_{2}\right)}^{\text{T}}$ is the wavevector. The matrix ***T*** is given by ***T*** = (***t***^(1)^, ***t***^(2)^).

The equations of motion of the chiral lattice can be written in the form [30,31]

2.2 $$[C-\omega^{2}(M-A)]U=\text{0},$$

where ***M*** = diag{*m*, *m*} is the mass matrix,

2.3 $$A=\left(\begin{array}{cc} 0 & -i\alpha \\ i\alpha & 0 \end{array}\right)$$

is the spinner matrix and

2.4 $$C=c\left(\begin{array}{cc} 3-2\cos(\zeta l+\xi l)-\frac{1}{2}[\cos(\zeta l)+\cos\left(\xi l\right)] & \frac{\sqrt{3}}{2}[\cos(\xi l)-\cos\left(\zeta l\right)]\\ \frac{\sqrt{3}}{2}[\cos(\xi l)-\cos\left(\zeta l\right)] & 3-\frac{3}{2}[\cos(\zeta l)+\cos\left(\xi l\right)] \end{array}\right)$$

is the stiffness matrix, where $\zeta =k_{1}/2+\sqrt{3}k_{2}/2$ and $\xi =k_{1}/2-\sqrt{3}k_{2}/2$.

We introduce the following normalizations:

2.5 $$\begin{array}{rl} & \tilde{x}=\frac{x}{l},\;\tilde{U}=\frac{U}{l},\;\tilde{u}=\frac{u}{l},\;\tilde{T}=\frac{T}{l},\;\tilde{k}=k\;l,\;\tilde{\zeta }=\zeta \;l,\;\tilde{\xi }=\xi \;l,\;\tilde{C}=\frac{C}{c},\\ \; & \tilde{M}=\frac{M}{m},\;\tilde{A}=\frac{A}{m},\;\tilde{\alpha }=\frac{\alpha }{m},\;\tilde{\omega }=\frac{\omega }{\sqrt{c/m}},\;\tilde{t}=t\sqrt{\frac{c}{m}}, \end{array}\}$$

where the quantities with the symbol ‘∼’ are dimensionless.

The frequency $\tilde{\omega }$ and the wavevector $\tilde{k}$ are related by the *dispersion relation* of the system, which is given by [30,31]

2.6 $$(1-{\tilde{\alpha }}^{2}){\tilde{\omega }}^{4}-\mathrm{tr}(\tilde{C})\;{\tilde{\omega }}^{2}+\det(\tilde{C})=0.$$

When $\tilde{\alpha }=1$, equation (2.6) degenerates and hence the dispersion diagrams include only one dispersion surface. This effect of degeneracy is discussed in detail in [30,31]. When $\tilde{\alpha }\neq 1$, the two positive solutions of the biquadratic equation in $\tilde{\omega }$ (2.6) are

2.7*a* $${\tilde{\omega }}^{\left(1\right)}(\tilde{k},\tilde{\alpha })=\sqrt{\frac{\mathrm{tr}(\tilde{C})-\sqrt{{\mathrm{tr}}^{2}(\tilde{C})-4(1-{\tilde{\alpha }}^{2})\det(\tilde{C})}}{2(1-{\tilde{\alpha }}^{2})}}$$

and

2.7*b* $${\tilde{\omega }}^{\left(2\right)}(\tilde{k},\tilde{\alpha })=\sqrt{\frac{\mathrm{tr}(\tilde{C})+\sqrt{{\mathrm{tr}}^{2}(\tilde{C})-4(1-{\tilde{\alpha }}^{2})\det(\tilde{C})}}{2(1-{\tilde{\alpha }}^{2})}}.$$

We note that ${\tilde{\omega }}^{\left(2\right)}$ takes imaginary values for $\tilde{\alpha }>1$. After calculating the eigenfrequencies ${\tilde{\omega }}^{\left(j\right)}$ for a certain wavevector, the corresponding eigenvectors ${\tilde{U}}^{\left(j\right)}={\tilde{U}}^{\left(j\right)}(\tilde{k},\tilde{\alpha })$ (*j* = 1, 2) can be determined analytically from (2.2).

**Figure 2.**
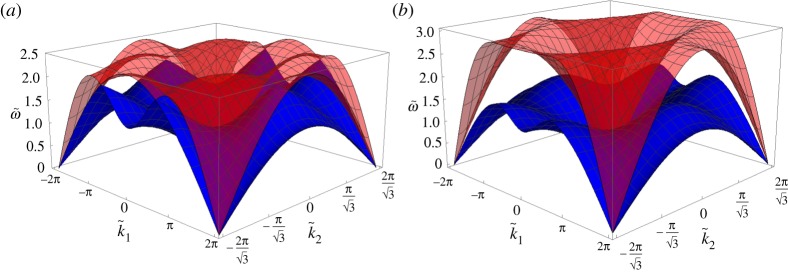
Lower and upper dispersion surfaces for the triangular lattice in figure 1, calculated for (*a*) $\tilde{\alpha }=0$ and (*b*) $\tilde{\alpha }=0.5$. (Online version in colour.)

The dispersion surfaces for $\tilde{\alpha }=0$ (non-chiral case) and $\tilde{\alpha }=0.5$ are presented in figure 2. The main effect of the gyroscopic spinners on the dispersion surfaces of the lattice is to decrease (or increase) the values of ${\tilde{\omega }}^{\left(1\right)}$ (or ${\tilde{\omega }}^{\left(2\right)}$) for a fixed wave vector $\tilde{k}$ [30,31]. For $\tilde{\alpha }=0$, the two dispersion surfaces touch at the Dirac points ${\left(k_{1},k_{2}\right)}^{\text{T}}={\left(\pm 4\pi /3,0\right)}^{\text{T}}$ and ${\left(k_{1},k_{2}\right)}^{\text{T}}={\left(\pm 2\pi /3,\pm 2\pi /\sqrt{3}\right)}^{\text{T}}$. For $\tilde{\alpha }>0$, the dispersion surfaces no longer touch so that the Dirac cones are ‘broken’.

### (b) Definitions of lattice flux and lattice circulation

As discussed in [21] for the non-chiral case ($\tilde{\alpha }=0$), waves propagating in a lattice can be characterized quantitatively by using the operators of *lattice flux* and *lattice circulation*. These are defined as (see [21])

2.8 $${\tilde{\Phi }}_{\tilde{u}}=i\frac{\sqrt{3}}{2}\;\tilde{u}\cdot \tilde{f}$$

and

2.9 $${\tilde{\Gamma }}_{\tilde{u}}=-i\frac{\sqrt{3}}{2}\;(\tilde{u}\times \tilde{f})\cdot e_{3},$$

respectively, where ***e***_3_ is the unit vector parallel to the *x*_3_-axis. In (2.8) and (2.9) we have also introduced the vector $\tilde{f}$, given by

2.10 $$\tilde{f}={\left(2\sin\left(\tilde{\zeta }+\tilde{\xi }\right)+\sin\left(\tilde{\zeta }\right)+\sin\left(\tilde{\xi }\right),\;\sqrt{3}[\sin\left(\tilde{\zeta }\right)-\sin\left(\tilde{\xi }\right)]\right)}^{T},$$

that depends on the geometry of the lattice.

In the long wavelength limit when $|\tilde{k}|\to 0$, the lattice approximates a continuum. In this limit, $\tilde{f}∼3\tilde{k}$. In a continuum, waves where the displacement $\tilde{u}$ is perpendicular (or parallel) to the wavevector $\tilde{k}$ are denoted as shear (or pressure) waves. Substituting $\tilde{f}=3\tilde{k}$ in (2.8) and (2.9), we notice that in a continuum shear (or pressure) waves correspond to flux-free (or circulation-free) waves. For intermediate and large values of the modulus of the wavevector, the continuum concepts of shear and pressure waves cannot be applied to the lattice. Instead, we will employ the definitions (2.8) and (2.9) to fully characterize waves propagating in the discrete system.

The vector $\tilde{u}$ in (2.8) and (2.9) represents the time-harmonic displacement of a lattice particle, calculated for a given eigenvector $\tilde{U}$. For $\tilde{\alpha }<1$, there are two eigenfrequencies and hence two eigenvectors $\tilde{U}$ for any value of the wavevector $\tilde{k}$. Denoting the coordinates of the central node of the lattice periodic cell shown in figure 1*c* as ${\tilde{x}}^{0}={\left(0,0\right)}^{T}$, the coordinates of the central node of the cell ***n*** ($n\in Z^{2}$) are $\tilde{x}={\tilde{x}}^{\left(n,0\right)}={\tilde{x}}^{0}+\tilde{T}n$. Using the Bloch–Floquet conditions (2.1), the time-harmonic displacement of a lattice particle for a given eigenvector is expressed by u~(j)(x~,t~)=Re(U~(j)(x~0)ei(ω~(j)t~+k~⋅T~n)) (*j* = 1, 2), where ${\tilde{U}}^{\left(j\right)}({\tilde{x}}^{0})e^{i({\tilde{\omega }}^{\left(j\right)}\tilde{t}+\tilde{k}\cdot \tilde{T}n)}={\tilde{u}}^{\left(j\right)}({\tilde{x}}^{0},\tilde{t})e^{i\tilde{k}\cdot \tilde{T}n}$ and ${\tilde{u}}^{\left(j\right)}({\tilde{x}}^{0},\tilde{t})$ is the displacement at ${\tilde{x}}^{0}$. We now concentrate on the displacement ${\tilde{u}}^{\left(j\right)}({\tilde{x}}^{0},\tilde{t})$, that can also be written as

2.11 u~(j)(x~0,t~)=(Re(U~1(j))cos⁡(ω~(j)t~)−Im(U~1(j))sin⁡(ω~(j)t~)Re(U~2(j))cos⁡(ω~(j)t~)−Im(U~2(j))sin⁡(ω~(j)t~)),j=1,2.

The trajectory of the particle is an ellipse, since the eigenvectors are complex. In the non-chiral case ($\tilde{\alpha }=0$) the particles trajectories are straight lines, since the eigenvectors are real. We also note that in the chiral lattice the eigenvectors are generally non-orthogonal. They satisfy the relation

2.12 $${\left(\;\bar{{\tilde{U}}^{\left(j\right)}}\right)}^{\text{T}}{\tilde{U}}^{\left(i\right)}+{\left(\;\bar{{\tilde{U}}^{\left(j\right)}}\right)}^{\text{T}}R{\tilde{U}}^{\left(i\right)}=0\;\left(i\neq j\right)\text{, with }R=\left(\begin{array}{cc} 0 & \text{i}\tilde{\alpha }\\ -\text{i}\tilde{\alpha } & 0 \end{array}\right),$$

which reduces to the orthogonality condition when $\tilde{\alpha }=0$.

## 3. Wave characterization in the chiral lattice

**Figure 3.**
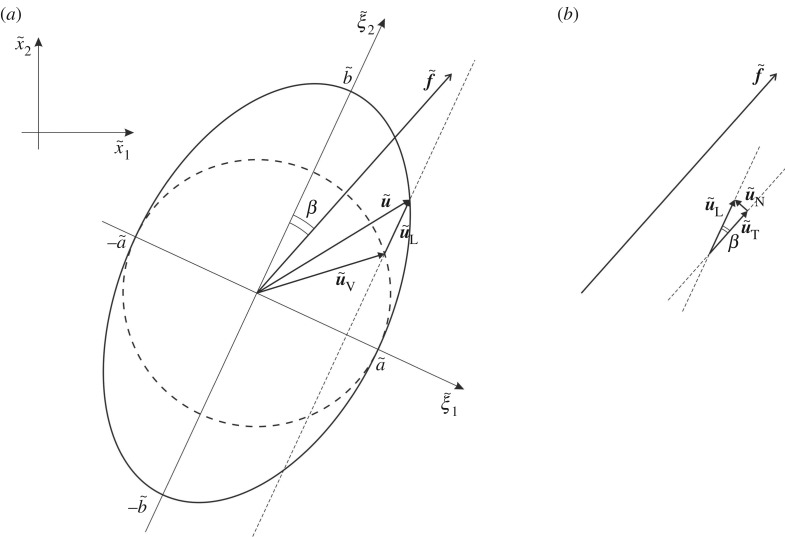
(*a*) Generic elliptical trajectory of a lattice particle in the chiral lattice and decomposition of the displacement field $\tilde{u}$ into a vortex component ${\tilde{u}}_{\text{V}}$ (corresponding to circular motion) and a straight-line component ${\tilde{u}}_{\text{L}}$ (corresponding to a straight-line motion parallel to the major axis of the ellipse); (*b*) a secondary decomposition of the straight-line field ${\tilde{u}}_{\text{L}}$ into a component ${\tilde{u}}_{\text{T}}$ tangential to $\tilde{f}$ and a component ${\tilde{u}}_{\text{N}}$ normal to $\tilde{f}$.

As discussed in §2b, each particle in the chiral lattice describes an elliptical trajectory, shown in figure 3*a*. The lengths of the minor and major semi-axes of the ellipse are denoted by $\tilde{a}$ and $\tilde{b}$, respectively. The angle between the major axis of the ellipse and the vector $\tilde{f}$ is denoted by *β*. We note that *β* is identical to the angle between the straight trajectory of a particle in the non-chiral case ($\tilde{\alpha }=0$) and the vector $\tilde{f}$. As $\tilde{\alpha }$ decreases, the resulting ellipses become narrower with the major axis direction remaining fixed. In the limit when $\tilde{\alpha }\to 0$ we retrieve the straight-line motion (in the direction of the major axis) we observe in the corresponding non-chiral case ($\tilde{\alpha }=0$).

### (a) Decomposition of the displacement field

As shown in figure 3*a*, the displacement $\tilde{u}$ can be decomposed into a component parallel to the major axis of the ellipse, denoted as ${\tilde{u}}_{\text{L}}$, and a component ${\tilde{u}}_{\text{V}}$ whose end describes a circular trajectory. The subscript ‘L’ in ${\tilde{u}}_{\text{L}}$ stands for line, since a particle having that displacement would move in a straight line parallel to the major axis. The subscript ‘V’ in ${\tilde{u}}_{\text{V}}$ stands for vortex, since it corresponds to a circular trajectory.

The straight-line component ${\tilde{u}}_{\text{L}}$ can be further decomposed into a component ${\tilde{u}}_{\text{T}}$ parallel to $\tilde{f}$, characterized by zero circulation, and a component ${\tilde{u}}_{\text{N}}$ perpendicular to $\tilde{f}$, having zero flux (figure 3*b*). This secondary decomposition was also used in [21] to characterize waves in the non-chiral case ($\tilde{\alpha }=0$), where the displacement $\tilde{u}={\tilde{u}}_{\text{L}}$ (${\tilde{u}}_{\text{V}}=\text{0}$). Therefore, the displacement field in the chiral lattice $\tilde{u}={\tilde{u}}_{\text{L}}+{\tilde{u}}_{\text{V}}$ consists of a ‘non-chiral’ component ${\tilde{u}}_{\text{L}}$ and a ‘chiral’ component ${\tilde{u}}_{\text{V}}$. Both ${\tilde{u}}_{\text{L}}$ and ${\tilde{u}}_{\text{V}}$ are functions of the wave number $\tilde{k}$ and the spinner constant $\tilde{\alpha }$.

It is important to note that the decomposition of the displacement field is not unique and the decomposition introduced above emphasizes and distils the circular (or vortex) displacement field, associated with chiral motion. One such alternative is to decompose the displacement field into components perpendicular and parallel to the major axis of the elliptical, chiral displacement. In this alternative decomposition, however, the two components would include the vortex motion, and a comparison with the non-chiral case ($\tilde{\alpha }=0$) would be less straightforward.

The degree of chirality in a lattice with gyroscopic spinners can be measured by the following parameter:

3.1 $$\chi^{\left(j\right)}=\frac{{\tilde{a}}^{\left(j\right)}}{{\tilde{b}}^{\left(j\right)}},\;j=1,2,$$

which represents the ratio of the length of the minor semi-axis to the length of the major semi-axis of the ellipse. We note that 0 ≤ *χ*^(*j*)^ ≤ 1, where the lower limit *χ*^(*j*)^ = 0 is found in the non-chiral case ($\tilde{\alpha }=0$) where the trajectory is always a straight line, while the upper limit *χ*^(*j*)^ = 1 is reached in the chiral lattice in some special cases when the trajectory is a circle (vortex waveforms).

The lengths of the minor and major semi-axes can be determined from the eigenvectors calculated from the dispersive properties of the system. Using (2.11), the canonical equation for the ellipse can be written as

3.2 $$\frac{1}{\det(B^{\left(j\right)})}[B_{11}^{\left(j\right)}{\tilde{x}}_{1}^{2}-2B_{12}^{\left(j\right)}{\tilde{x}}_{1}{\tilde{x}}_{2}+B_{22}^{\left(j\right)}{\tilde{x}}_{2}^{2}]=1,$$

where the components of the matrix ***B***^(*j*)^ = (***B***^(*j*)^)^T^ (*j* = 1, 2) are

3.3*a* $$B_{11}^{\left(j\right)}=\text{Re}{\left({\tilde{U}}_{2}^{\left(j\right)}\right)}^{2}+\text{Im}{\left({\tilde{U}}_{2}^{\left(j\right)}\right)}^{2},$$

3.3*b* B12(j)=B21(j)=−[Re(U~1(j))Re(U~2(j))+Im(U~1(j))Im(U~2(j))]

3.3*c* $$\text{and }B_{22}^{\left(j\right)}=\text{Re}{\left({\tilde{U}}_{1}^{\left(j\right)}\right)}^{2}+\text{Im}{\left({\tilde{U}}_{1}^{\left(j\right)}\right)}^{2}.$$

The eigenvalues of ***B***^(*j*)^ are given by

3.4 $$\lambda_{\pm }^{\left(j\right)}=\frac{\mathrm{tr}(B^{\left(j\right)})\pm \sqrt{{\mathrm{tr}}^{2}(B^{\left(j\right)})-4\det(B^{\left(j\right)})}}{2\det(B^{\left(j\right)})},$$

while the eigenvectors of ***B***^(*j*)^ are expressed by

3.5 $$V_{\pm }^{\left(j\right)}=\left(\begin{array}{c} \frac{B_{11}^{\left(j\right)}-B_{22}^{\left(j\right)}\pm \sqrt{{\mathrm{tr}}^{2}(B^{\left(j\right)})-4\det(B^{\left(j\right)})}}{-2B_{12}^{\left(j\right)}}\\ 1 \end{array}\right).$$

The lengths of the minor and major semi-axes of the ellipse are then given by

3.6 $${\tilde{a}}^{\left(j\right)}=\frac{1}{\sqrt{\lambda_{+}^{\left(j\right)}}}\;\text{and}\;{\tilde{b}}^{\left(j\right)}=\frac{1}{\sqrt{\lambda_{-}^{\left(j\right)}}}.$$

The direction of the major axis is defined by $V_{-}^{\left(j\right)}$. The angle *β*^(*j*)^ (*j* = 1, 2) is the angle between the major axis of the ellipse and the vector $\tilde{f}$. In this paper, we take *β*^(*j*)^ as the acute angle between $V_{-}^{\left(j\right)}$ and $\tilde{f}$, such that 0 ≤ *β*^(*j*)^ ≤ *π*/2:

3.7*a* $$\beta^{\left(j\right)}=\arccos\left(\frac{V_{-}^{\left(j\right)}\cdot \tilde{f}}{|\tilde{f}||{\tilde{V}}_{-}^{\left(j\right)}|}\right)\;\text{if}\;V_{-}^{\left(j\right)}\cdot \tilde{f}>0$$

and

3.7*b* $$\beta^{\left(j\right)}=\pi -\arccos\left(\frac{V_{-}^{\left(j\right)}\cdot \tilde{f}}{|\tilde{f}||{\tilde{V}}_{-}^{\left(j\right)}|}\right)\;\text{if}\;V_{-}^{\left(j\right)}\cdot \tilde{f}<0.$$

### (b) Flux and circulation in the chiral lattice

As shown in (2.8) and (2.9), the flux and circulation are pure imaginary quantities, with moduli $|{\tilde{\Phi }}_{\tilde{u}}|$ and $|{\tilde{\Gamma }}_{\tilde{u}}|$, respectively. In this paper, we denote by $\|{\tilde{\Phi }}_{\tilde{u}}\|=\max|{\tilde{\Phi }}_{\tilde{u}}|$ and $\|{\tilde{\Gamma }}_{\tilde{u}}\|=\max|{\tilde{\Gamma }}_{\tilde{u}}|$ the ‘amplitudes’ of flux and circulation, respectively.

As discussed in [21], in the non-chiral case ($\tilde{\alpha }=0$) the flux amplitude for the lower dispersion surface $\left\|{\tilde{\Phi }}_{\tilde{u}}^{\left(1\right)}\right\|$ is equal to the circulation amplitude for the upper dispersion surface $\left\|{\tilde{\Gamma }}_{\tilde{u}}^{\left(2\right)}\right\|$, if the eigenvectors corresponding to the two dispersion surfaces are normalized to have the same modulus; furthermore, $\left\|{\tilde{\Phi }}_{\tilde{u}}^{\left(2\right)}\|=\|{\tilde{\Gamma }}_{\tilde{u}}^{\left(1\right)}\right\|$. This is due to the orthogonality of the eigenvectors in the non-chiral case (see (2.12) for $\tilde{\alpha }=0$), as discussed in [21]. In the chiral lattice generally these relations do not hold, since the eigenvectors are not orthogonal.

**Figure 4.**
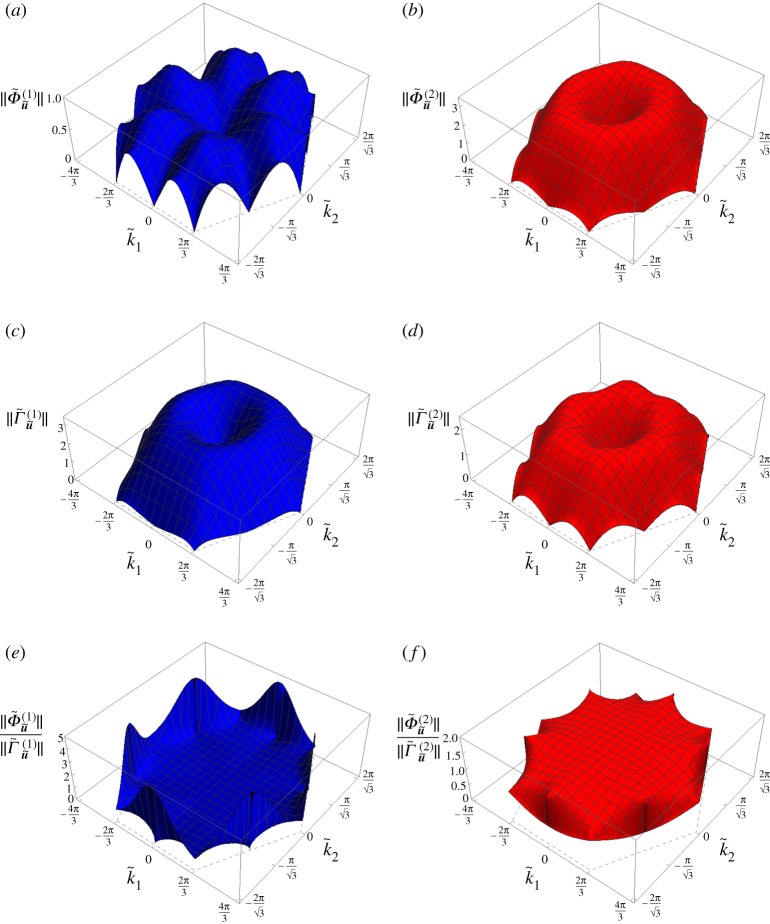
The amplitudes (*a*) $\left\|{\tilde{\Phi }}_{\tilde{u}}^{\left(1\right)}\right\|$, (*b*) $\left\|{\tilde{\Phi }}_{\tilde{u}}^{\left(2\right)}\right\|$, (*c*) $\left\|{\tilde{\Gamma }}_{\tilde{u}}^{\left(1\right)}\right\|$, (*d*) $\left\|{\tilde{\Gamma }}_{\tilde{u}}^{\left(2\right)}\right\|$, and the ratios (*e*) $\left\|{\tilde{\Phi }}_{\tilde{u}}^{\left(1\right)}\|/\|{\tilde{\Gamma }}_{\tilde{u}}^{\left(1\right)}\right\|$, (*f* ) $\left\|{\tilde{\Phi }}_{\tilde{u}}^{\left(2\right)}\|/\|{\tilde{\Gamma }}_{\tilde{u}}^{\left(2\right)}\right\|$ in the first Brillouin zone, calculated for the chiral lattice with $\tilde{\alpha }=0.5$. (Online version in colour.)

**Figure 5.**
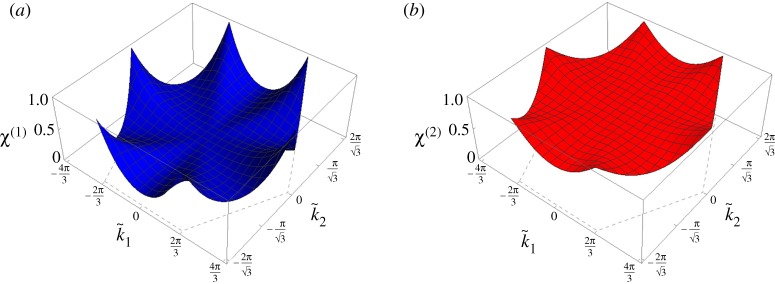
Ratios (*a*) *χ*^(1)^ and (*b*) *χ*^(2)^ in the first Brillouin zone, calculated for the chiral lattice with $\tilde{\alpha }=0.5$. (Online version in colour.)

The three-dimensional representations in the $\tilde{k}$-plane of the amplitudes of flux and circulation for both dispersion surfaces are plotted in figure 4*a*–*d* for a representative value of the spinner constant $\tilde{\alpha }=0.5$. The plots are limited to the first Brillouin zone, defined as the hexagon connecting the points D in figure 8. The qualitative features shown in figure 4*a*–*d* persist for all values of $\tilde{\alpha }$ for the lower dispersion surface and for $0<\tilde{\alpha }<1$ for the upper dispersion surface. The angles *β*^(1)^ and *β*^(2)^ are the same as those found in the non-chiral case (see figs 5c and 5d in [21]).

We point out that the maps of flux and circulation in the $\tilde{k}$-plane depend on the chosen normalization of the eigenvectors. In the computations presented in this paper, the eigenvectors in (2.11) are normalized such that $\tilde{b}=1$. This is in agreement with the normalization adopted in [21] for the non-chiral case ($\tilde{\alpha }=0$), whereby the maximum straight-line displacement of each lattice particle is 1. However, it is important to note that the ratio of flux to circulation for each dispersion surface is independent of the normalization of the eigenvectors. The ratio gives a measure, independent of the normalization of the eigenvectors, of the relative contributions of flux and circulation for a given wave. In figure 4*e*,*f* we show the three-dimensional representations of the ratios $\left\|{\tilde{\Phi }}_{\tilde{u}}^{\left(1\right)}\|/\|{\tilde{\Gamma }}_{\tilde{u}}^{\left(1\right)}\right\|$ and $\left\|{\tilde{\Phi }}_{\tilde{u}}^{\left(2\right)}\|/\|{\tilde{\Gamma }}_{\tilde{u}}^{\left(2\right)}\right\|$, respectively. In particular, we observe that in the long wavelength limit circulation (or flux) is dominant on the lower (or upper) surface.

From figure 4*a*–*d* it can be noted that the amplitudes of flux and circulation are continuous functions of $\tilde{k}$. From the figures, it is also apparent that there are no lines where either the flux or the circulation are zero, while in the non-chiral case ($\tilde{\alpha }=0$), as observed in [21], there are special lines in the $\tilde{k}$-plane where waves are either flux-free or circulation-free, even for large values of $|\tilde{k}|$.

In figure 5*a*,*b*, we show *χ*^(1)^ and *χ*^(2)^ as functions of the wavevector. Interestingly, *χ*^(2)^ ≥ *χ*^(1)^ for any value of the wavevector. In addition, from figure 5*a*,*b*, we observe that *χ*^(2)^ = *χ*^(1)^ = 1 at all the points D in figure 8. At these points, every particle in the chiral lattice moves in a circle. In the non-chiral case ($\tilde{\alpha }=0$), points D are vertices of Dirac cones.

**Figure 6.**
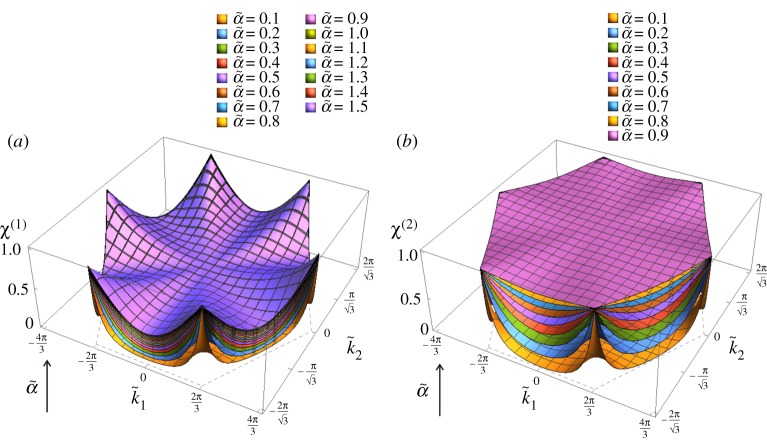
(*a*) *χ*^(1)^ and (*b*) *χ*^(2)^ in the first Brillouin zone, determined for different values of $\tilde{\alpha }$, as indicated in the legends. (Online version in colour.)

Figure 6*a*,*b* shows *χ*^(1)^ and *χ*^(2)^ in the $\tilde{k}$-plane for different values of $\tilde{\alpha }$. We notice that both *χ*^(1)^ and *χ*^(2)^ increase with the spinner constant for any value of the wavevector. We have checked this analytically by verifying that $\partial \chi^{\left(j\right)}/\partial \tilde{\alpha }>0$ (*j* = 1, 2) for any $\tilde{k}$ and for any $\tilde{\alpha }$ (the results are not included here for brevity).

The flux and circulation of the total displacement field $\tilde{u}$ can be decomposed into two components, one associated with the vortex field ${\tilde{u}}_{\text{V}}$ and the other with the straight-line field ${\tilde{u}}_{\text{L}}$. The straight-line displacement can be further decomposed into a circulation-free and a flux-free component. Referring to figure 3*a*, the total displacement can be written in the rotated frame aligned with the principal axes $\left({\tilde{\xi }}_{1},{\tilde{\xi }}_{2}\right)$ of the ellipse as

3.8 $$\tilde{u}={\tilde{u}}_{\text{V}}+{\tilde{u}}_{\text{L}}=\left(\begin{array}{c} \tilde{a}\cos\left(\tilde{\omega }\tilde{t}\right)\\ \tilde{a}\sin\left(\tilde{\omega }\tilde{t}\right) \end{array}\right)+\left(\begin{array}{c} 0\\ (\tilde{b}-\tilde{a})\sin\left(\tilde{\omega }\tilde{t}\right) \end{array}\right).$$

Using (2.8) and (2.9), we find that the flux and circulation associated with the vortex field are given by

3.9 $${\tilde{\Phi }}_{{\tilde{u}}_{\text{V}}}=i\frac{\sqrt{3}}{2}\tilde{a}|\tilde{f}|\sin\left(\tilde{\omega }\tilde{t}+\beta \right)\;\mathrm{and}\;{\tilde{\Gamma }}_{{\tilde{u}}_{\text{V}}}=i\frac{\sqrt{3}}{2}\tilde{a}|\tilde{f}|\cos\left(\tilde{\omega }\tilde{t}+\beta \right),$$

respectively. Accordingly, the flux and circulation of the vortex field differ in phase by *π*/2 and have the same amplitude, namely

3.10 $$\|{\tilde{\Phi }}_{{\tilde{u}}_{\text{V}}}\|=\|{\tilde{\Gamma }}_{{\tilde{u}}_{\text{V}}}\|=\frac{\sqrt{3}}{2}\tilde{a}|\tilde{f}|.$$

Such a vortex field possesses the following properties:

— the trajectories of nodal points within the lattice are circular, with a phase shift present between different elementary cells;
— the maximum amplitudes of lattice flux and lattice circulation are equal.

This is a third fundamental field present in characterizing waves in chiral lattices, in addition to the flux-free and circulation-free fields observed in non-chiral case ($\tilde{\alpha }=0$), as discussed in [21].

The straight-line displacement field can be decomposed into a component tangential to $\tilde{f}$ and a component normal to $\tilde{f}$, such that ${\tilde{u}}_{\text{L}}={\tilde{u}}_{\text{T}}+{\tilde{u}}_{\text{N}}$ (figure 3*b*). The tangential component ${\tilde{u}}_{\text{T}}$ has zero circulation, while its flux is equal to

3.11 $${\tilde{\Phi }}_{{\tilde{u}}_{\text{T}}}=i\frac{\sqrt{3}}{2}(\tilde{b}-\tilde{a})|\tilde{f}|\cos\left(\beta \right)\sin\left(\tilde{\omega }\tilde{t}\right),$$

with amplitude

3.12 $$\|{\tilde{\Phi }}_{{\tilde{u}}_{\text{T}}}\|=\frac{\sqrt{3}}{2}(\tilde{b}-\tilde{a})|\tilde{f}|\cos\left(\beta \right).$$

On the other hand, the normal component ${\tilde{u}}_{\text{N}}$ is characterized by zero flux and non-zero circulation, given by

3.13 $${\tilde{\Gamma }}_{{\tilde{u}}_{\text{N}}}=-i\frac{\sqrt{3}}{2}(\tilde{b}-\tilde{a})|\tilde{f}|\sin\left(\beta \right)\sin\left(\tilde{\omega }\tilde{t}\right),$$

having amplitude

3.14 $$\|{\tilde{\Gamma }}_{{\tilde{u}}_{\text{N}}}\|=\frac{\sqrt{3}}{2}(\tilde{b}-\tilde{a})|\tilde{f}|\sin\left(\beta \right).$$

**Figure 7.**
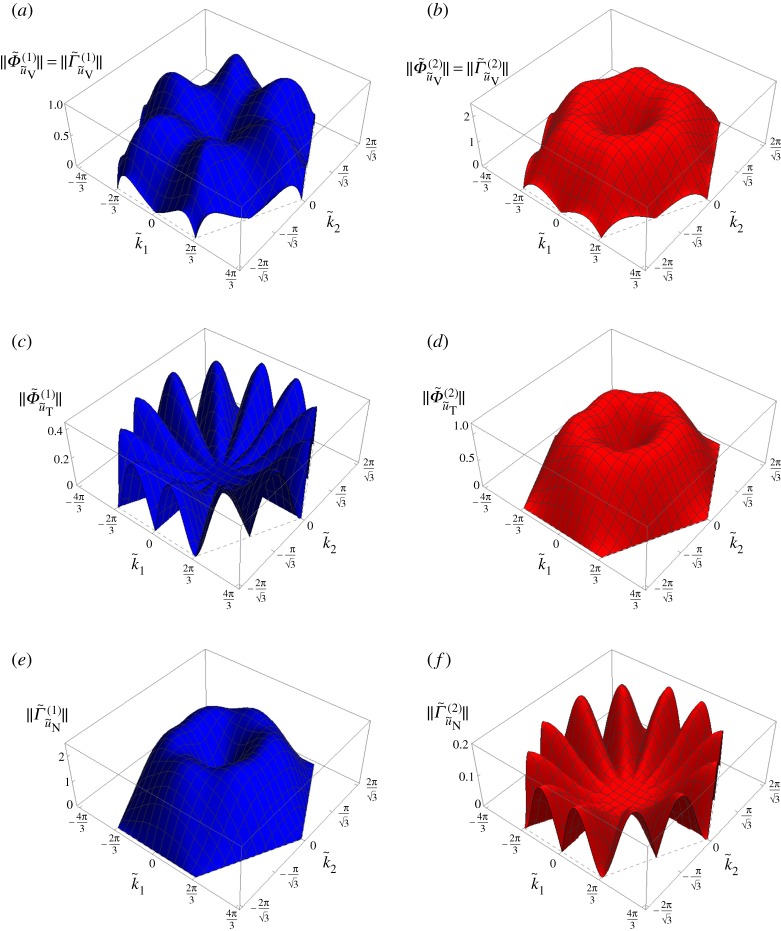
Non-zero amplitudes of flux and circulation associated with (*a*,*b*) the vortex component ${\tilde{u}}_{\text{V}}$, (*c*,*d*) straight-line tangential component ${\tilde{u}}_{\text{T}}$ and (*e*,*f* ) straight-line normal component ${\tilde{u}}_{\text{N}}$ in the first Brillouin zone. The value of the spinner constant $\tilde{\alpha }=0.5$ is the same as in figure 4. (Online version in colour.)

The amplitudes of flux and circulation for the displacement components ${\tilde{u}}_{\text{V}}$, ${\tilde{u}}_{\text{T}}$ and ${\tilde{u}}_{\text{N}}$ as functions of the wavevector $\tilde{k}$ are presented in figure 7. The same normalization of the eigenvectors as for the diagrams in figure 4 has been used, namely ${\tilde{b}}^{\left(j\right)}=1$ (*j* = 1, 2).

**Figure 8.**
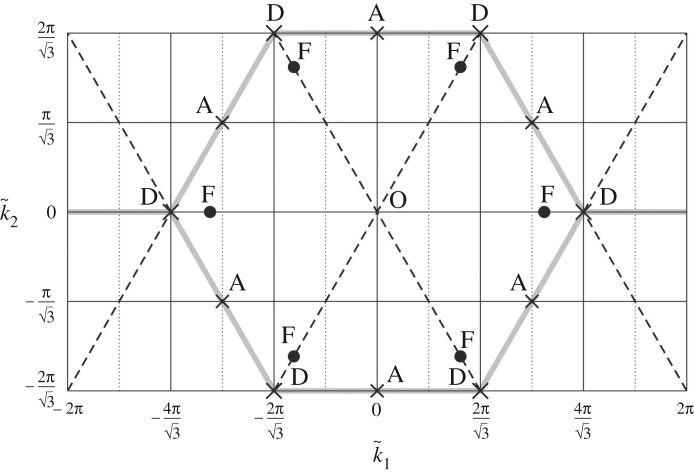
Stationary points of the lower dispersion surface (A, D) and upper dispersion surface (A, D, F), whose properties are detailed in tables 1–4, are shown in the extended rectangular domain which includes the hexagonal cell in the reciprocal space. The crosses represent fixed points, while the dots indicate points whose positions vary with $\tilde{\alpha }$. In the figure, the positions of points F are given for $\tilde{\alpha }=0$.

Comparing figure 7*a* and *b*, we note that the contribution of the vortex component to the total displacement is larger for the upper surface. This in agreement with the diagrams in figure 5*a*,*b*, whereby *χ*^(2)^ ≥ *χ*^(1)^ and hence the radius of the circular trajectory for the upper surface is larger than that for the lower surface (${\tilde{a}}^{\left(2\right)}\geq {\tilde{a}}^{\left(1\right)}$) keeping the length of the major semi-axis the same (${\tilde{b}}^{\left(2\right)}={\tilde{b}}^{\left(1\right)}=1$). Concerning the straight-line component of the displacement, figure 7*c*–*f* reveals that the flux and circulation in the chiral lattice have features similar to those identified in the non-chiral case ($\tilde{\alpha }=0$) [21]. In particular, $\|{\tilde{\Phi }}_{{\tilde{u}}_{\text{T}}}^{\left(1\right)}\|=\|{\tilde{\Gamma }}_{{\tilde{u}}_{\text{N}}}^{\left(2\right)}\|=0$ in the lines given by $\arctan\left({\tilde{k}}_{2}/{\tilde{k}}_{1}\right)=(n-1)\pi /6$ with *n* = 1, …, 12, while $\|{\tilde{\Gamma }}_{{\tilde{u}}_{\text{N}}}^{\left(1\right)}\|=\|{\tilde{\Phi }}_{{\tilde{u}}_{\text{T}}}^{\left(2\right)}\|=0$ in the hexagon connecting the points D in figure 8. Additionally, we note that the ratio of the flux to the circulation for the lower surface ($\left\|{\tilde{\Phi }}_{{\tilde{u}}_{\text{T}}}^{\left(1\right)}\|/\|{\tilde{\Gamma }}_{{\tilde{u}}_{\text{N}}}^{\left(1\right)}\right\|$) is generally smaller than that for the upper surface (($\left\|{\tilde{\Phi }}_{{\tilde{u}}_{\text{T}}}^{\left(2\right)}\|/\|{\tilde{\Gamma }}_{{\tilde{u}}_{\text{N}}}^{\left(2\right)}\right\|$)). This means that for $\tilde{\alpha }=0.5$ the straight-line component of the displacement is of flux-free type for the lower surface and of circulation-free type for the upper surface. However, differently from the non-chiral case ($\tilde{\alpha }=0$), here the contribution of the vortex component (characterized by equal amplitudes of flux and circulation) is significant in that it changes the overall motion of the lattice.

The amplitudes of flux and circulation of the total displacement $\tilde{u}$, shown in figure 4, can be obtained in terms of the amplitudes of flux and circulation of the displacement components ${\tilde{u}}_{\text{V}}$, ${\tilde{u}}_{\text{T}}$ and ${\tilde{u}}_{\text{N}}$, presented in figure 7, as follows:

3.15*a* $$\|{\tilde{\Phi }}_{\tilde{u}}\|=\sqrt{\|{\tilde{\Phi }}_{{\tilde{u}}_{\text{V}}}{\|}^{2}+\|{\tilde{\Phi }}_{{\tilde{u}}_{\text{T}}}{\|}^{2}+2\|{\tilde{\Phi }}_{{\tilde{u}}_{\text{V}}}\|\|{\tilde{\Phi }}_{{\tilde{u}}_{\text{T}}}\|\cos\left(\beta \right)}$$

and

3.15*b* $$\|{\tilde{\Gamma }}_{\tilde{u}}\|=\sqrt{\|{\tilde{\Gamma }}_{{\tilde{u}}_{\text{V}}}{\|}^{2}+\|{\tilde{\Gamma }}_{{\tilde{u}}_{\text{N}}}{\|}^{2}+2\|{\tilde{\Gamma }}_{{\tilde{u}}_{\text{V}}}\|\|{\tilde{\Gamma }}_{{\tilde{u}}_{\text{N}}}\|\sin\left(\beta \right)}.$$

The ratio of the length of the minor semi-axis to the length of the major semi-axis of the ellipse can also be expressed as a function of the flux and circulation of the displacement components:

3.16 $$\chi =\frac{\left\|{\tilde{\Phi }}_{{\tilde{u}}_{\text{V}}}\right\|}{\|{\tilde{\Phi }}_{{\tilde{u}}_{\text{V}}}\|+\sqrt{\|{\tilde{\Phi }}_{{\tilde{u}}_{\text{T}}}{\|}^{2}+\|{\tilde{\Gamma }}_{{\tilde{u}}_{\text{N}}}{\|}^{2}}}.$$

### (c) Wave propagation at the stationary points of the dispersion surfaces

The stationary points of the two dispersion surfaces of the chiral lattice are shown in figure 8. The properties of the stationary points of the lower dispersion surface are detailed in table 1 for any value of the spinner constant $\tilde{\alpha }$, while those of the upper dispersion surface are given in tables 2, 3 and 4 for different ranges of $\tilde{\alpha }$. The coordinates of only one instance of each type of stationary point are detailed in tables 1–4; the coordinates of the other corresponding stationary points can be obtained by rotating the given coordinates by *nπ*/3 (*n* = 1, 2, …, 5) with respect to the origin O.

**Table 1.** Stationary points of the lower dispersion surface for $0<\tilde{\alpha }<\infty$.

point	$\left({\tilde{k}}_{1},{\tilde{k}}_{2}\right)$	${\tilde{\omega }}_{1}$	type
A	$\left(\pi ,\frac{\pi }{\sqrt{3}}\right)$	$\sqrt{\frac{6}{2+\sqrt{1+3{\tilde{\alpha }}^{2}}}}$	saddle point
D	$\left(\frac{4\pi }{3},0\right)$	$\sqrt{\frac{9}{2(1+\tilde{\alpha })}}$	maximum

**Table 2.** Stationary points of the upper dispersion surface for $0<\tilde{\alpha }<1/3$.

point	$\left({\tilde{k}}_{1},{\tilde{k}}_{2}\right)$	${\tilde{\omega }}_{2}$	type
A	$\left(\pi ,\frac{\pi }{\sqrt{3}}\right)$	$\sqrt{\frac{6}{2-\sqrt{1+3{\tilde{\alpha }}^{2}}}}$	maximum
D	$\left(\frac{4\pi }{3},0\right)$	$\sqrt{\frac{9}{2(1-\tilde{\alpha })}}$	minimum
F	$\left(4\arccos\left[\frac{\sqrt{7-27{\tilde{\alpha }}^{2}}}{4}\right],0\right)$	$\frac{9\sqrt{1+3{\tilde{\alpha }}^{2}}}{4}$	saddle point

**Table 3.** Stationary points of the upper dispersion surface for $1/3<\tilde{\alpha }<\sqrt{7/27}$.

point	$\left({\tilde{k}}_{1},{\tilde{k}}_{2}\right)$	${\tilde{\omega }}_{2}$	type
A	$\left(\pi ,\frac{\pi }{\sqrt{3}}\right)$	$\sqrt{\frac{6}{2-\sqrt{1+3{\tilde{\alpha }}^{2}}}}$	maximum
D	$\left(\frac{4\pi }{3},0\right)$	$\sqrt{\frac{9}{2(1-\tilde{\alpha })}}$	maximum
F	$\left(2\pi -\arccos\left[\frac{-1-27{\tilde{\alpha }}^{2}}{8}\right],\sqrt{3}\arccos\left[\frac{-1-27{\tilde{\alpha }}^{2}}{8}\right]-\frac{2\pi }{\sqrt{3}}\right)$	$\frac{9\sqrt{1+3{\tilde{\alpha }}^{2}}}{4}$	saddle point

**Table 4.** Stationary points of the upper dispersion surface for $\sqrt{7/27}<\tilde{\alpha }<1$.

point	$\left({\tilde{k}}_{1},{\tilde{k}}_{2}\right)$	${\tilde{\omega }}_{2}$	type
A	$\left(\pi ,\frac{\pi }{\sqrt{3}}\right)$	$\sqrt{\frac{6}{2-\sqrt{1+3{\tilde{\alpha }}^{2}}}}$	saddle point
D	$\left(\frac{4\pi }{3},0\right)$	$\sqrt{\frac{9}{2(1-\tilde{\alpha })}}$	maximum
F	$\left(\pi ,\frac{\pi }{\sqrt{3}}\right)$	$\frac{9\sqrt{1+3{\tilde{\alpha }}^{2}}}{4}$	saddle point

It is interesting to note that points A and D do not change their positions in the $\tilde{k}$-plane as $\tilde{\alpha }$ is changed, while positions of points F (which are stationary points only for the upper surface) are $\tilde{\alpha }$ dependent. In particular, for $\tilde{\alpha }=0$, points F occupy the positions shown in figure 8. When $\tilde{\alpha }=1/3$, they coincide with points D; hence, for the upper dispersion surface, points D are minima for $\tilde{\alpha }<1/3$, saddle points for $\tilde{\alpha }=1/3$ and maxima for $1/3<\tilde{\alpha }<1$. When $\tilde{\alpha }\geq \sqrt{7/27}$, points F coincide with points A; accordingly, for the upper dispersion surface, points A are maxima for $\tilde{\alpha }<\sqrt{7/27}$ and become saddle points for $\sqrt{7/27}\leq \tilde{\alpha }<1$. Therefore, $\tilde{\alpha }=1/3$ and $\tilde{\alpha }=\sqrt{7/27}$ are special values of the spinner constant, for which the response of the lattice changes significantly in terms of dynamic anisotropy.

The frequency of each stationary point varies with the spinner constant $\tilde{\alpha }$. The type of stationary point on the upper dispersion surface is also dependent on the spinner constant.

While in the non-chiral case ($\tilde{\alpha }=0$) points F were on special lines characterized by either zero flux or zero circulation (see fig. 7*b* in [21]), in the chiral case both the flux and circulation at points F are generally different from zero. Conversely, points A and D are characterized by zero flux and zero circulation, since $\tilde{f}=\text{0}$ at these points. Additionally, $\left\|{\tilde{\Phi }}_{\tilde{u}}^{\left(1\right)}\|/\|{\tilde{\Gamma }}_{\tilde{u}}^{\left(1\right)}\|<1<\|{\tilde{\Phi }}_{\tilde{u}}^{\left(2\right)}\|/\|{\tilde{\Gamma }}_{\tilde{u}}^{\left(2\right)}\right\|$ at points A, while $\|{\tilde{\Phi }}_{\tilde{u}}^{\left(1\right)}\|/\|{\tilde{\Gamma }}_{\tilde{u}}^{\left(1\right)}\|,\|{\tilde{\Phi }}_{\tilde{u}}^{\left(2\right)}\|/\|{\tilde{\Gamma }}_{\tilde{u}}^{\left(2\right)}\|\to 1$ at points D. Therefore, *χ*^(*j*)^ = 1 (*j* = 1, 2) at points D (see also figure 5*a*,*b*) and hence the corresponding motion of each lattice particle is circular.

## 4. Illustrative examples and physical interpretation of wave characterization

In this section, we investigate how waves propagate in the chiral medium for different values of the wavevector. In particular, we show the total displacement field of the lattice in time, as well as the motion of the lattice particles when a single component of the displacement field (vortex, straight-line, straight-line tangential or straight-line normal) is considered. In the calculations, the spinner constant is taken as $\tilde{\alpha }=0.5$. We emphasize that increasing $\tilde{\alpha }$ augments the contribution of the vortex component to the total field and makes the elliptical trajectories of the lattice particles less eccentric.

**Figure 9.**
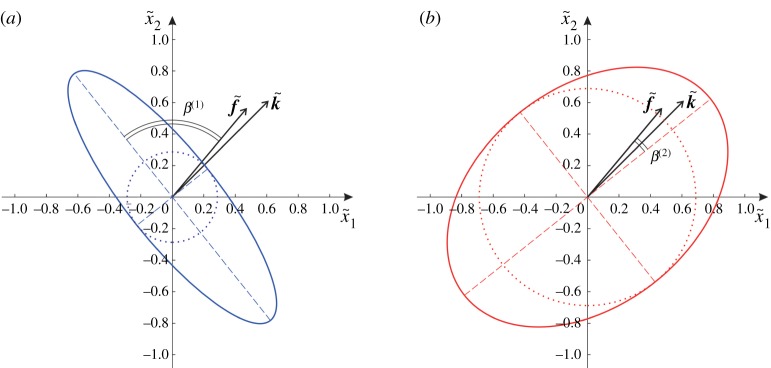
Trajectory of a generic particle in the chiral lattice with spinner constant $\tilde{\alpha }=0.5$ corresponding to the (*a*) lower and (*b*) upper dispersion surface, calculated for ${\tilde{k}}_{1}=2$ and ${\tilde{k}}_{2}=2$. The dotted circles correspond to the vortex components of the displacements. (Online version in colour.)

Firstly, we consider a relatively large value of $|\tilde{k}|$, namely ${\tilde{k}}_{1}=2$ and ${\tilde{k}}_{2}=2$. The vector $\tilde{f}$, defined in (2.10), is given by $\tilde{f}={\left(1.548,1.847\right)}^{T}$. The corresponding frequencies for the lower and upper dispersion surface are ${\tilde{\omega }}^{\left(1\right)}=1.360$ and ${\tilde{\omega }}^{\left(2\right)}=2.779$, respectively. The angles between the vector $\tilde{f}$ and the major axes of the elliptical trajectories of the lattice particles for the two dispersion surfaces are *β*^(1)^ = 1.370 and *β*^(2)^ = 0.200. We remark that, because of the choice of decomposition of the displacement, the angles *β*^(*j*)^ (*j* = 1, 2) do not vary with $\tilde{\alpha }$; changing the value of $\tilde{\alpha }$ changes only the vortex component. The amplitudes of flux and circulation corresponding to the lower surface are given by $\|{\tilde{\Phi }}_{\tilde{u}}^{\left(1\right)}\|=0.720$ and $\|{\tilde{\Gamma }}_{\tilde{u}}^{\left(1\right)}\|=2.049$, while those obtained for the upper surface are $\|{\tilde{\Phi }}_{\tilde{u}}^{\left(2\right)}\|=2.066$ and $\|{\tilde{\Gamma }}_{\tilde{u}}^{\left(2\right)}\|=1.468$. The ratios of flux to circulation are respectively $\|{\tilde{\Phi }}_{\tilde{u}}^{\left(1\right)}\|/\|{\tilde{\Gamma }}_{\tilde{u}}^{\left(1\right)}\|=0.351$ and $\|{\tilde{\Phi }}_{\tilde{u}}^{\left(2\right)}\|/\|{\tilde{\Gamma }}_{\tilde{u}}^{\left(2\right)}\|=1.407$. The values above show that waves in this chiral system are a mixture of flux-free, circulation-free and vortex contributions. The ratios between the lengths of the minor semi-axes to the lengths of the major semi-axes of the ellipse for the lower and upper surface are *χ*^(1)^ = 0.287 and *χ*^(2)^ = 0.688, respectively. These values of *χ*^(*j*)^ are associated with the amplitudes of flux and circulation of the vortex motion, which are $\|{\tilde{\Phi }}_{{\tilde{u}}_{\text{V}}}^{\left(1\right)}\|=\|{\tilde{\Gamma }}_{{\tilde{u}}_{\text{V}}}^{\left(1\right)}\|=0.600$ for the lower dispersion surface and $\|{\tilde{\Phi }}_{{\tilde{u}}_{\text{V}}}^{\left(2\right)}\|=\|{\tilde{\Gamma }}_{{\tilde{u}}_{\text{V}}}^{\left(2\right)}\|=1.437$ for the upper dispersion surface. The flux and circulation corresponding to the straight-line component are $\|{\tilde{\Phi }}_{{\tilde{u}}_{\text{T}}}^{\left(1\right)}\|=0.296$, $\|{\tilde{\Gamma }}_{{\tilde{u}}_{\text{N}}}^{\left(1\right)}\|=1.458$ for the lower surface and $\|{\tilde{\Phi }}_{{\tilde{u}}_{\text{T}}}^{\left(2\right)}\|=0.637$, $\|{\tilde{\Gamma }}_{{\tilde{u}}_{\text{N}}}^{\left(2\right)}\|=0.129$ for the upper surface. The elliptical trajectory of each lattice particle is illustrated in figure 9*a* and figure 9*b* for the lower and upper dispersion surface, respectively. The circles in figure 9 correspond to the vortex components of the displacement (compare with figure 3).

Video 1a and 1f in the electronic supplementary material show the total time-dependent displacement fields corresponding to the two dispersion surfaces, determined for the wavevector with components ${\tilde{k}}_{1}=2$ and ${\tilde{k}}_{2}=2$. The contributions due to the vortex components are illustrated in video 1b and 1g in the electronic supplementary material, while those due to the straight-line components are displayed in video 1c and 1h in the electronic supplementary material. The straight-line field is further decomposed into a flux-free (normal) motion (see video 1d and 1i in the electronic supplementary material) and a circulation-free (tangential) motion (see video 1e and 1j in the electronic supplementary material). In the videos, the amplitude of each displacement component is proportional to its contribution to the total displacement. Accordingly, it can be observed that for the upper surface the vortex component plays a significant role for this choice of parameters. In the videos, the vectors $\tilde{k}$ and $\tilde{f}$ are indicated in magenta and green, respectively, and the trajectories of the particles are plotted in red.

**Figure 10.**
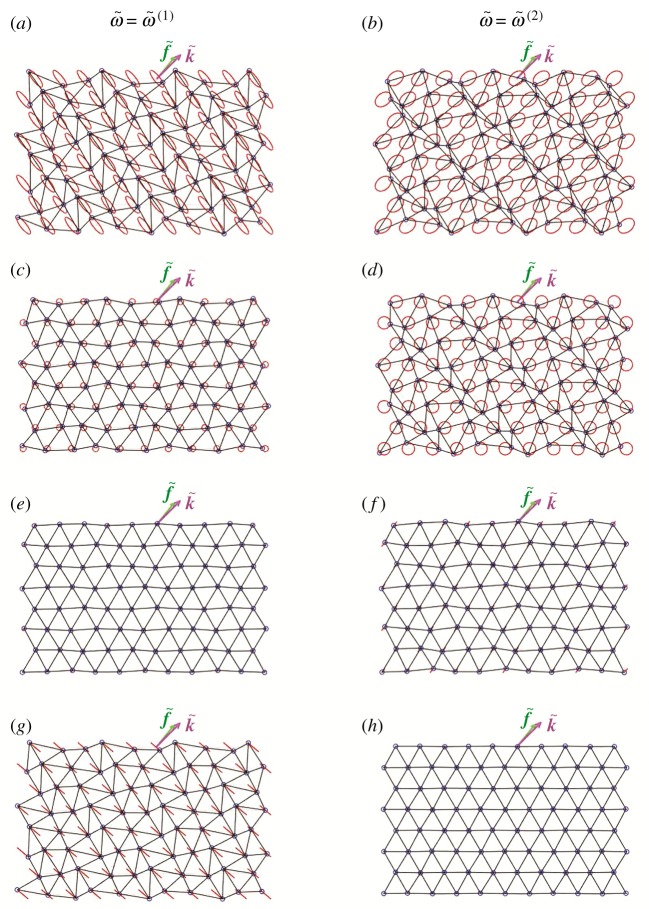
Displacement field in the chiral lattice for the (*a*,*c*,*e*,*g*) lower and (*b*,*d*,*f* ,*h*) upper dispersion surface, calculated for ${\tilde{k}}_{1}=2$, ${\tilde{k}}_{2}=2$ and $\tilde{\alpha }=0.5$. (*a*,*b*) Total displacements, (*c*,*d*) vortex components, (*e*,*f* ) straight-line tangential components, (*g*,*h*) straight-line normal components. The vectors $\tilde{k}$ and $\tilde{f}$ are also plotted. The trajectories of the lattice particles are shown in red. (Online version in colour.)

Figure 10*a* and 10*b* show instantaneous snapshots of the displacement fields for ${\tilde{k}}_{1}=2$, ${\tilde{k}}_{2}=2$ calculated for the lower and upper dispersion surface, respectively. The corresponding vortex components are presented in figure 10*c*,*d*, the straight-line tangential components in figure 10*e*,*f* and the straight-line normal components in figure 10*g*,*h*.

**Figure 11.**
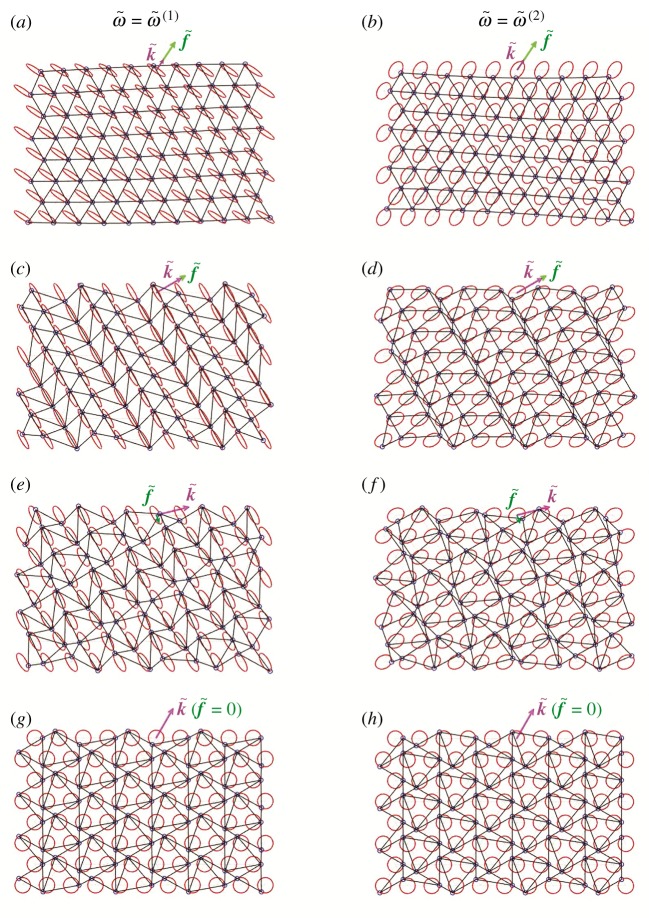
Displacements fields in the chiral lattice with spinner constant $\tilde{\alpha }=0.5$ relative to the (*a*,*c*,*e*,*g*) lower and (*b*,*d*,*f* ,*h*) upper dispersion surface, calculated for different values of the wavevector: (*a*,*b*) ${\tilde{k}}_{1}=0.200$, ${\tilde{k}}_{2}=0.297$; (*c*,*d*) ${\tilde{k}}_{1}=2.150$, ${\tilde{k}}_{2}=1.241$; (*e*,*f* ) ${\tilde{k}}_{1}=3.665$, ${\tilde{k}}_{2}=0.907$; (*g*,*h*) ${\tilde{k}}_{1}=2\pi /3$, ${\tilde{k}}_{2}=2\pi /\sqrt{3}$. (Online version in colour.)

Video 2a–2j in the electronic supplementary material illustrate how waves propagate in the chiral lattice when the wavevector is ${\left({\tilde{k}}_{1},{\tilde{k}}_{2}\right)}^{\text{T}}={\left(0.200,0.297\right)}^{\text{T}}$. This wavevector has a modulus that is significantly smaller than that considered in video 1a–1f in the electronic supplementary material. The displacement fields in the lattice are also shown in figure 11*a* and 11*b* for the lower and upper dispersion surface, respectively. From video 2d (or video 2j) in the electronic supplementary material, we observe that the tangential (or normal) component of the straight-line motion is negligibly small for the lower (or upper) dispersion surface. Consequently, when $|\tilde{k}|\to 0$, only two displacement components are significant: the vortex motion and the straight-line normal (or straight-line tangential) motion for the lower (or upper) dispersion surface. This is consistent with the long wavelength limit behaviour in the non-chiral case ($\tilde{\alpha }=0$), except that here ($\tilde{\alpha }=0.5$) there is an additional vortex waveform component.

Similar observations can be made in the scenario where $\tilde{k}$ has a large modulus, but is taken on one of the lines where either flux or circulation of the straight-line field is null. For example, video 3a–3j in the electronic supplementary material are obtained when $\tilde{k}={\left(2.150,1.241\right)}^{\text{T}}$, which lies on the line inclined by 30^°^ to the ${\tilde{k}}_{1}$-axis. In this case, the straight-line motion for the lower (or upper) dispersion surface is flux-free (or circulation-free) because the straight-line displacement is perpendicular (or parallel) to $\tilde{f}$. This shows consistency with the behaviour observed in [21], where for $\tilde{\alpha }=0$ there are radial lines from the origin in the $\tilde{k}$-plane along which there are pure flux-free or circulation-free waves. Again, here we have similar behaviour with an additional vortex waveform component. The total displacement fields for the lower and upper dispersion surface are also illustrated in figure 11*c* and 11*d*, respectively.

Video 4a–4j in the electronic supplementary material are produced for $\tilde{k}={\left(3.665,0.907\right)}^{\text{T}}$, which belongs to the perimeter of the hexagon connecting points D in figure 8 (see also figure 11*e*,*f* ). Since for the straight-line motion the points on the sides of this hexagon are circulation-free (or flux-free) for the lower (or upper) surface, this motion consists of only the component parallel (or perpendicular) to $\tilde{f}$. Similar behaviour was found in [21] in the non-chiral case ($\tilde{\alpha }=0$), except that here ($\tilde{\alpha }=0.5$) there is an additional contribution to the displacement field due to the vortex waveform.

Finally, video 5a–5j in the electronic supplementary material show the lattice motion corresponding to one of the stationary points D in figure 8, namely ${\left({\tilde{k}}_{1},{\tilde{k}}_{2}\right)}^{\text{T}}={\left(2\pi /3,2\pi /\sqrt{3}\right)}^{\text{T}}$ (see also figures 11*g*,*h*). In this case, each particle of the lattice describes a circular trajectory, for both dispersion surfaces. This is in agreement with the results in figure 5*a*,*b*, where *χ*^(*j*)^ = 1 (*j* = 1, 2). This is a special case where pure vortex waveforms occur in the chiral lattice. The points D are located at the values of the $\tilde{k}$ vector which in the non-chiral case ($\tilde{\alpha }=0$) would correspond to the Dirac points.

We note that the rotations of the lattice particles corresponding to the two dispersion surfaces are always in opposite directions.

## 5. Dynamic degeneracy in chiral elastic systems

In this section, we study the factor *χ*^(*j*)^ for the lower (*j* = 1) and upper (*j* = 2) dispersion surface for some limit cases of the spinner constant $\tilde{\alpha }$. In addition, we discuss the possibility of creating vortex waveforms for any value of the wavevector when $\tilde{\alpha }$ tends to unity.

### (a) Lower dispersion surface

Consider the long wavelength limit, when $|\tilde{k}|\to 0$. In this limit, expression (3.16) leads to

5.1 $$\begin{array}{rl} \chi^{\left(1\right)} & ∼\sqrt{\frac{2+(1+\sqrt{1+3{\tilde{\alpha }}^{2}})/({\tilde{\alpha }}^{2})-\sqrt{\left({\tilde{\alpha }}^{4}+2\left(1+\sqrt{1+3{\tilde{\alpha }}^{2}}\right)+{\tilde{\alpha }}^{2}\left(5+2\sqrt{1+3{\tilde{\alpha }}^{2}}\right))/({\tilde{\alpha }}^{4}\right)}}{2+(1+\sqrt{1+3{\tilde{\alpha }}^{2}})/({\tilde{\alpha }}^{2})+\sqrt{\left({\tilde{\alpha }}^{4}+2\left(1+\sqrt{1+3{\tilde{\alpha }}^{2}}\right)+{\tilde{\alpha }}^{2}\left(5+2\sqrt{1+3{\tilde{\alpha }}^{2}}\right))/({\tilde{\alpha }}^{4}\right)}}}\\ & \;\text{when}\;|\tilde{k}|\to 0. \end{array}$$

**Figure 12.**
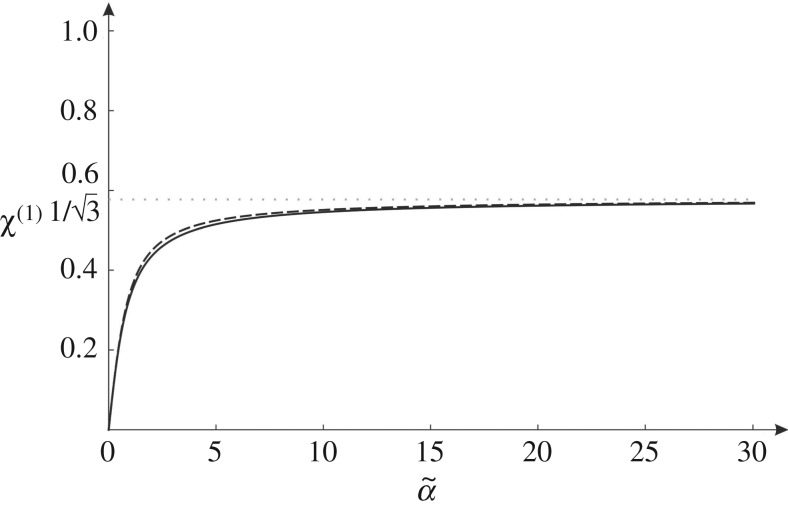
For the long wavelength limit, graph of *χ*^(1)^ versus $\tilde{\alpha }$ (solid line) together with its approximation (5.2) (dashed line).

The function above is shown in figure 12 by a solid line.

Since $\chi^{\left(1\right)}∼\tilde{\alpha }/2$ when $\tilde{\alpha }\to 0$ and $\chi^{\left(1\right)}\to 1/\sqrt{3}$ when $\tilde{\alpha }\to \infty$, the expression (5.1) can be approximated by the simpler function

5.2 $$\chi^{\left(1\right)}\approx \frac{2}{\sqrt{3}\pi }\arctan\left(\frac{\sqrt{3}\pi }{4}\tilde{\alpha }\right),$$

which is represented by a dashed line in figure 12.

The limit $\chi^{\left(1\right)}\to 1/\sqrt{3}$ when $\tilde{\alpha }\to \infty$ can be proved as follows. For simplicity and without loss of generality, we take again Re(U~2(1))=1 and Im(U~2(1))=0 in (2.11). The frequency ${\tilde{\omega }}^{\left(1\right)}$, corresponding to the lower dispersion surface and given by (2.7a), has the following asymptotic expansion for large values of the spinner constant and in the long wavelength limit:

5.3 $${\tilde{\omega }}^{\left(1\right)}∼\frac{\sqrt[{4}]{27}}{2\sqrt{2}}\left(\frac{{\tilde{k}}_{1}^{2}}{2{\tilde{k}}_{2}}+{\tilde{k}}_{2}\right)\sqrt{\epsilon }\;\text{when }|\tilde{k}|\to 0\;\text{and}\;\epsilon =\frac{1}{\tilde{\alpha }}\to 0.$$

Substituting the above expression into equation (2.2) to determine the eigenvectors, we find that Re(U~1(1)) and Im(U~1(1)) in (2.11) are

5.4 Re(U~1(1))∼−2k~1k~23k~12+k~22, Im(U~1(1))∼3(k~12+k~22)3k~12+k~22when |k~|→0 and ϵ=1α~→0.

The eigenvalues (3.4) are found to be

5.5 $$\lambda_{-}^{\left(1\right)}∼\frac{3{\tilde{k}}_{1}^{2}+{\tilde{k}}_{2}^{2}}{3({\tilde{k}}_{1}^{2}+{\tilde{k}}_{2}^{2})},\;\lambda_{+}^{\left(1\right)}∼\frac{3{\tilde{k}}_{1}^{2}+{\tilde{k}}_{2}^{2}}{{\tilde{k}}_{1}^{2}+{\tilde{k}}_{2}^{2}}\;\text{when}\;|\tilde{k}|\to 0\;\text{and}\;\epsilon =\frac{1}{\tilde{\alpha }}\to 0.$$

Hence, equation (3.1) leads to

5.6 $$\chi^{\left(1\right)}=\sqrt{\frac{\lambda_{-}^{\left(1\right)}}{\lambda_{+}^{\left(1\right)}}}∼\frac{1}{\sqrt{3}}\;\text{when }|\tilde{k}|\to 0\;\text{and}\;\epsilon =\frac{1}{\tilde{\alpha }}\to 0.$$

The same limit for $\tilde{\alpha }\to \infty$ is attained by the approximation (5.2).

For large values of the wavevector, in the limit when $\tilde{\alpha }\to \infty$, *χ*^(1)^ is given by

5.7 $$\chi^{\left(1\right)}=\chi^{\left(1\right)}({\tilde{k}}_{1},{\tilde{k}}_{2})∼\sqrt{\frac{1+N_{1}/D^{2}-N_{2}/D}{1+N_{1}/D^{2}+N_{2}/D}}\;\text{when}\;\tilde{\alpha }\to \infty ,$$

where

5.8*a* $$\begin{array}{rl} & N_{1}=12(5-4c_{1}^{2}-6c_{1}c_{2}+c_{1}^{2}c_{2}^{2}+4c_{1}^{3}c_{2}), \end{array}$$

5.8*b* $$\begin{array}{rl} & N_{2}=4\sqrt{4-7c_{1}^{2}+4c_{1}^{4}+2c_{1}c_{2}-3c_{2}^{2}+4c_{1}^{2}c_{2}^{2}-4c_{1}^{3}c_{2}} \end{array}$$

5.8*c* $$\begin{array}{rl} \;\text{and}\; & D=10-8c_{1}^{2}-2c_{1}c_{2}, \end{array}$$

with $c_{1}=\cos({\tilde{k}}_{1}/2)$ and $c_{2}=\cos(\sqrt{3}{\tilde{k}}_{2}/2)$. We note that, in the unit cell, D=0 for ${\left({\tilde{k}}_{1},{\tilde{k}}_{2}\right)}^{\text{T}}={\left(0,0\right)}^{\text{T}}$ and ${\left({\tilde{k}}_{1},{\tilde{k}}_{2}\right)}^{\text{T}}={\left(\pm 2\pi ,\pm 2\pi /\sqrt{3}\right)}^{\text{T}}$. Nonetheless, $N_{1}/D^{2}\to 1$ and $N_{2}/D\to 1$ for ${\left({\tilde{k}}_{1},{\tilde{k}}_{2}\right)}^{\text{T}}\to {\left(0,0\right)}^{\text{T}}$ and ${\left({\tilde{k}}_{1},{\tilde{k}}_{2}\right)}^{\text{T}}\to {\left(\pm 2\pi ,\pm 2\pi /\sqrt{3}\right)}^{\text{T}}$.

**Figure 13.**
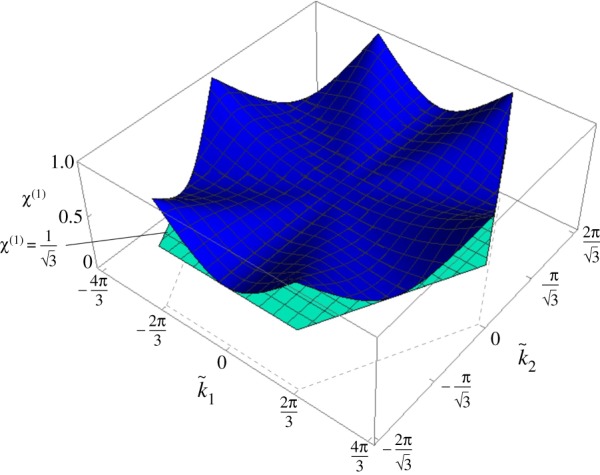
*χ*^(1)^ in the limit when $\tilde{\alpha }\to \infty$. The plane $\chi^{\left(1\right)}=1/\sqrt{3}$ is also included in the figure. Both surfaces are limited to the first Brillouin zone. (Online version in colour.)

The function $\chi^{\left(1\right)}({\tilde{k}}_{1},{\tilde{k}}_{2})$ given in (5.7) is shown in figure 13. We observe that the global minimum of the function is $1/\sqrt{3}$, that is the value obtained for $|\tilde{k}|\to 0$ (see equation (5.6)). Furthermore, $\chi^{\left(1\right)}=1/\sqrt{3}$ along the radials given by $\arctan\left({\tilde{k}}_{2}/{\tilde{k}}_{1}\right)=(2n-1)\pi /6$ with *n* = 1, …, 6. The global maxima are found at the points D in figure 8, where *χ*^(1)^ = 1.

### (b) Upper dispersion surface

As in §5a, we first analyse the case when $|\tilde{k}|\to 0$. Using (3.16), we find that

5.9 $$\begin{array}{rl} \chi^{\left(1\right)} & ∼\sqrt{\frac{6+\left(1+\sqrt{1+3{\tilde{\alpha }}^{2}}\right)/({\tilde{\alpha }}^{2})-\sqrt{\left(9{\tilde{\alpha }}^{4}+2\left(1+\sqrt{1+3{\tilde{\alpha }}^{2}}\right)-3{\tilde{\alpha }}^{2}\left(1+2\sqrt{1+3{\tilde{\alpha }}^{2}}\right)\right)/({\tilde{\alpha }}^{4})}}{6+\left(1+\sqrt{1+3{\tilde{\alpha }}^{2}}\right)/({\tilde{\alpha }}^{2})+\sqrt{\left(9{\tilde{\alpha }}^{4}+2\left(1+\sqrt{1+3{\tilde{\alpha }}^{2}}\right)-3{\tilde{\alpha }}^{2}\left(1+2\sqrt{1+3{\tilde{\alpha }}^{2}}\right)\right)/({\tilde{\alpha }}^{4})}}}\\ & \;\text{when }|\tilde{k}|\to 0. \end{array}$$

**Figure 14.**
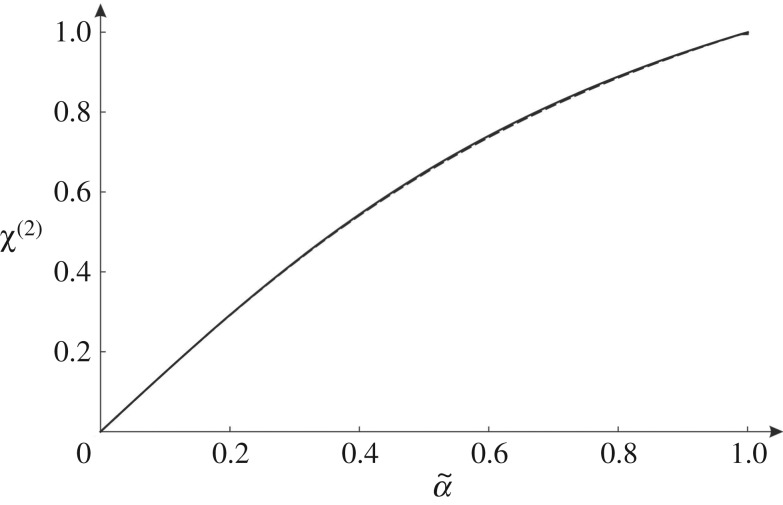
For $|\tilde{k}|\to 0$, *χ*^(2)^ versus $\tilde{\alpha }$ (solid line), compared with the analytical approximation (5.10) (dashed line).

The function above is shown in figure 14 by a solid line. In this case $0\leq \tilde{\alpha }<1$, since ${\tilde{\omega }}^{\left(2\right)}$ takes imaginary values for $\tilde{\alpha }>1$ (see equation (2.7bb)).

In figure 14, the dashed line represents the following analytical approximation of *χ*^(2)^:

5.10 $$\chi^{\left(2\right)}\approx C\arctan\left(\frac{3}{2C}\tilde{\alpha }\right),$$

where C is the root of the equation

5.11 $$C\arctan\left(\frac{3}{2C}\right)=1,$$

which gives C≈1.0337. The approximation (5.10) has the same limits as the function (5.9) for small and large values of $\tilde{\alpha }$, namely $3\tilde{\alpha }/2$ when $\tilde{\alpha }\to 0$ and 1 when $\tilde{\alpha }\to 1$.

We note that, as discussed below, for any $\tilde{k}$ (not only in the long wavelength limit) we have

5.12 $$\lim_{\tilde{\alpha }\to 1}\chi^{\left(2\right)}=1.$$

The frequency on the upper dispersion surface has the following asymptotic approximation when $\tilde{\alpha }\to 1$:

5.13 $${\tilde{\omega }}^{\left(2\right)}∼\frac{\sqrt{3-\cos\left({\tilde{k}}_{1}\right)-2\cos\left({\tilde{k}}_{1}/2\right)\cos\left(\sqrt{3}{\tilde{k}}_{2}/2\right)}}{\sqrt{\epsilon }}\;\text{for}\;\tilde{\alpha }=1-\epsilon \;\text{with}\;\epsilon \to 0^{+}.$$

Substituting the expression above into equation (2.2) and normalizing the eigenvector in (2.11) by setting Re(U~2(2))=1 and Im(U~2(2))=0, we obtain

5.14 Re(U~1(2))→0, Im(U~1(2))→1for α~=1−ϵ with ϵ→0+.

Consequently, from (3.4) $\lambda_{-}^{\left(2\right)}$ and $\lambda_{+}^{\left(2\right)}$ are found to be equal, and hence from (3.1) we have

5.15 $$\chi^{\left(2\right)}=\sqrt{\frac{\lambda_{-}^{\left(2\right)}}{\lambda_{+}^{\left(2\right)}}}\to 1\;\text{for}\;\tilde{\alpha }=1-\epsilon \;\text{with}\;\epsilon \to 0^{+}.$$

The results of this section demonstrate that pure vortex waveforms can be realized for any value of the wavevector at higher frequencies, when the spinner constant tends to its critical value ($\tilde{\alpha }\to 1$).

## 6. Conclusion

In this paper, we have demonstrated that the analytical concepts of lattice flux and lattice circulation represent canonical characteristics to describe polarization of waves in a chiral elastic lattice. This is especially important when the wavelength is comparable with the size of the elementary cell of the periodic system, where the continuum notions of pressure and shear waves cannot be used.

The procedure discussed in this paper allows for a canonical decomposition of a general waveform in a chiral lattice. Besides flux-free and circulation-free straight-line displacement patterns, typical of the non-chiral case ($\tilde{\alpha }=0$) discussed in [21], in a chiral lattice the concept of vortex waveforms has been introduced and investigated here.

As demonstrated in [21], the notion of pressure and shear waves in isotropic homogeneous continuous media allows for a generalization to elastic lattice systems in the context of lattice flux-free and lattice circulation-free waveforms. In the present paper, we advance further and use a new class of vortex waveforms, specifically for chiral elastic systems. In this context, there is an advantage in the representation including decomposition of waveforms into chiral and non-chiral components.

Typical time-harmonic patterns of motion of nodal points in the chiral elastic lattice are elliptical trajectories. Asymptotic analysis and animations have shown limit situations when vortex waveforms become dominant. In these cases, the trajectories of the lattice particles are circular and the amplitudes of lattice flux and lattice circulation are equal.

The analytical findings presented in this paper provide a new insight in the design and construction of numerical algorithms for the analysis of chiral elastic lattices. In particular, illustrative examples shown in figures 10 and 11 represent the displacement fields in the triangular lattice for different values of the wavevector $\tilde{k}$, and show a decomposition of waveforms into vortex, lattice flux-free and lattice circulation-free components. The effect of the spinner constant $\tilde{\alpha }$ and the wavevector $\tilde{k}$ on eccentricity of elliptical trajectories of lattice particles is shown in figure 6, which includes three-dimensional surface diagrams representing parameters *χ*^(1)^ and *χ*^(2)^.

This work allows for many extensions to heterogeneous lattices and lattices of other geometries. The vortex waveforms are expected to persist in other types of chiral elastic lattices.

## Supplementary Material

Description of the files in the Supplementary Material

## Supplementary Material

Video 1

## Supplementary Material

Video 2

## Supplementary Material

Video 3

## Supplementary Material

Video 4

## Supplementary Material

Video 5

## Data accessibility

The paper contains no experimental data. All results and illustrative computations are directly reproducible.
