# Supplementary material for: Wave polarization and dynamic degeneracy in a chiral elastic lattice
Source: Proc Math Phys Eng Sci. 2019 Dec 18;475(2232):20190313. doi: 10.1098/rspa.2019.0313 (PMC6936612; doi:10.1098/rspa.2019.0313)
Supplement: Description of the files in the Supplementary Material [file rspa20190313supp1.pdf]

Description of the files in the Supplementary Material

- "Video1a"-"Video1j" show the motions of an elastic lattice for the wave vector $\left(\tilde{k}_1, \tilde{k}_2\right)^{\mathrm{T}} = \left(2, 2\right)^{\mathrm{T}}$ at the frequencies of the lower and upper dispersion surfaces. The total displacement fields and their components (vortex, straight-line tangential and straight-line normal) are illustrated, as indicated in the names of the files.

- "Video2a"-"Video2j" show the motions of an elastic lattice for the wave vector $\left(\tilde{k}_1, \tilde{k}_2\right)^{\mathrm{T}} = \left(0.200, 0.297\right)^{\mathrm{T}}$ at the frequencies of the lower and upper dispersion surfaces. The total displacement fields and their components (vortex, straight-line tangential and straight-line normal) are illustrated, as indicated in the names of the files.

- "Video3a"-"Video3j" show the motions of an elastic lattice for the wave vector $\left(\tilde{k}_1, \tilde{k}_2\right)^{\mathrm{T}} = \left(2.150, 1.241\right)^{\mathrm{T}}$ at the frequencies of the lower and upper dispersion surfaces. The total displacement fields and their components (vortex, straight-line tangential and straight-line normal) are illustrated, as indicated in the names of the files.

- "Video4a"-"Video4j" show the motions of an elastic lattice for the wave vector $\left(\tilde{k}_1, \tilde{k}_2\right)^{\mathrm{T}} = \left(3.665, 0.907\right)^{\mathrm{T}}$ at the frequencies of the lower and upper dispersion surfaces. The total displacement fields and their components (vortex, straight-line tangential and straight-line normal) are illustrated, as indicated in the names of the files.

- "Video5a"-"Video5j" show the motions of an elastic lattice for the wave vector $\left(\tilde{k}_1, \tilde{k}_2\right)^{\mathrm{T}} = \left(2\pi/3, 2\pi/\sqrt{3}\right)^{\mathrm{T}}$ at the frequencies of the lower and upper dispersion surfaces. The total displacement fields and their components (vortex, straight-line tangential and straight-line normal) are illustrated, as indicated in the names of the files.
